# VR-Enabled Digital Twin Framework for Real-Time Wind Turbine Fault Detection and Sustainability Monitoring

**DOI:** 10.3390/s26206415

**Published:** 2026-10-10

**Authors:** Huma Tehreem, Muhammad Zahid Iqbal, Olugbenga Akinade

**Affiliations:** School of Computing, Engineering & Digital Technologies, Teesside University, Middlesbrough TS1 3BX, UK

**Keywords:** digital twin, fault detection, net-zero, real-time data synchronisation, sustainable energy management, wind turbine fault detection

## Abstract

The importance of wind turbine reliability for the performance and cost-effectiveness of wind power systems cannot be overstated, and advanced wind turbine condition monitoring is crucial to the solution. Providing effective condition monitoring for wind turbines is still difficult, as there is not a lot of insight in real time, nor is there any integrated sustainability assessment. While some solutions can be offered by digital twins, most existing solutions are not immersive, and do not merge performance information with environmental information. In this paper, an integrated VR-based Digital Twin framework for real-time simulation and condition monitoring as well as deterministic fault-risk assessment of wind turbines is presented. The model is developed in MATLAB using historical UK wind data and includes a reduced-order physics-based turbine model, aerodynamic behaviour, rotor dynamics, thermal modelling, controlled fault injection, and a healthy-reference/fault-exposed dual-turbine configuration. The temperature and vibration results are aggregated into a Fault-Risk Score (FRS) using predefined warning and fault thresholds, while a Health Index (HI), energy production and consequence metrics related to CO_2_ are complementary indicators of turbine condition and performance. The results indicate that there is a significant difference between the healthy turbine and the fault-exposed turbine, where the fault-exposed turbine has a higher health-index degradation, lower energy production, and higher fault-risk score. By the end of the simulation period, the turbine’s health index was reduced from >80% to 38% under fault conditions, leading to a 5% penalty in energy generation and a 5.1% elevation in the environmental ${\mathrm{CO}}_{2}$ impact metric. Additionally, the implementation using MATLAB–Unity showed real-time telemetry transmission and immersive visualisation at 72–90 FPS from the communication response time of less than 50 milliseconds. The system therefore provides an integrated platform for physics-based condition monitoring, controlled fault-risk assessment, sustainability consequence evaluation, and immersive operator visualisation, while offering a basis for future individual components and hardware-oriented validation.

## 1. Introduction

Wind power is a vital component of the global energy transition, offering a low carbon, scalable, and more secure alternative to electricity generation, and helping to mitigate fossil fuels’ contribution to emissions reduction goals [1]. According to the International Energy Agency, the world needs to install 390 GW of new wind power capacity every year until 2030 in order to meet global climate goals, as the levelised cost of wind energy drops, policy frameworks are put in place, and the need for decarbonisation grows [2,3]. As of 2024, the United Kingdom had approximately 14 GW of offshore and 15 GW of onshore wind capacity [4]. Wind power accounts for 20–25% of the UK’s electricity demand each year, and more than 50% during peak wind conditions, and the government aims for 50 GW of offshore wind to be installed by 2030 to reach the goal of net-zero by 2050 [5].

Despite such progress, wind power still has many hurdles to overcome in its operation [6]. Wind turbines operate under continuously varying environmental and loading conditions, including changes in wind speed and temperature [7]. In addition to humidity and salt spray conditions [8], which can contribute to component deterioration and unexpected downtime [9], offshore installations have to deal with challenging access, inspection, and maintenance conditions. For onshore turbines, operations and maintenance (O&M) costs account for 20–30% of the total life cycle costs and reach up to 35% in the case of offshore turbines [10]. The downtime of offshore wind turbines has been estimated to be in the range of £10,000 to £40,000 per day per turbine [11]. The same operational and maintenance issues are also found in offshore wind farms [12]. These challenges increase the importance of condition monitoring approaches that can identify abnormal operating behaviour before failures develop into costly unplanned downtime, while also supporting maintenance and operational decision-making.

Conventional condition monitoring uses Supervisory Control and Data Acquisition (SCADA) systems that are able to gather average operational data every 10 min to 1 h [13]. Maldonado-Correa et al. [14] have reviewed extensively the use of SCADA data for wind turbine condition monitoring. SCADA systems primarily provide aggregated operational measurements, such as power, wind speed, temperature, and rotational speed, depending on the turbine configuration and data-acquisition system. Dedicated condition-monitoring systems can additionally provide higher-resolution measurements such as vibration and component-specific temperature information. Typically, the analysis of SCADA data is done after data acquisition, making it difficult to detect faults in real time and possibly leading to a reactive maintenance approach [15]. In recent years, therefore, there has been an increasing interest in advanced fault detection techniques that would enable early detection of incipient faults in wind turbine systems [16]. Existing approaches can broadly be considered as signal-based, model-based, or data-driven methods. Signal-based approaches analyse measured quantities such as vibration, temperature, acoustic signals, or electrical characteristics; model-based approaches compare measured or estimated behaviour with a physical or mathematical reference model; and data-driven approaches learn patterns of normal and abnormal behaviour from historical data. Each approach provides useful information, but also involves trade-offs in terms of measurement requirements, model complexity, interpretability, and generalisation under changing operating conditions. Once anomalies are detected, significant damage can have taken place, necessitating emergency repairs, and extended downtimes, especially offshore where vessels can be weathered out. In multi-turbine configurations, standard 2D dashboards lose the spatial context, causing operator cognitive fatigue and delaying reaction times [17]. However, claims regarding improved operator performance require dedicated human-subject or usability validation; therefore, immersive visualisation should be considered an operator-support mechanism unless such validation is explicitly conducted. One major limitation of current research is the combination of immersive 3D visualisation and physics-based fault prediction [18]. The majority of the digital twin frameworks for power and energy systems are still confined to the static 2D environment or the post-processed simulation data [19]. These restrictions may hinder the ability to make timely decisions in maintenance and repair situations [20]. In addition, more sophisticated digital twin frameworks have been identified in recent reviews that are required to enable condition monitoring and predictive maintenance (PdM) applications in real-time [21]. Nevertheless, the integration of physics-based turbine modelling, multi-parameter condition indicators, immersive visualisation, and sustainability consequence metrics within a single digital-twin framework remains comparatively limited. There is a lack of research in the literature on integrating multi-parameter fault diagnostics with sustainability metrics.

Existing systems are based on single-parameter analyses (such as temperature or vibration analysis independently), which do not consider the multi-modal degradation [22]. Temperature and vibration should also be distinguished as different condition-indicator streams rather than assumed to represent the same physical measurement source. If thermal and vibrational stresses are combined, the health of a component cannot be accurately predicted from single-source data alone as described in [23]. Combining complementary thermal and vibration indicators can therefore provide a broader representation of abnormal turbine behaviour; however, such a combined indicator does not by itself establish component-specific fault isolation. Component-level diagnosis of, for example, main-shaft bearings, gearbox components, or generator bearings requires dedicated component models, appropriate measurements, and/or validated fault signatures. A major challenge is the lack of real-time CO_2_ emissions monitoring in current digital twin solutions [21]. In the context of a digital twin, however, CO_2_ should be distinguished from a directly measured diagnostic signal when it is calculated from energy production and an assumed carbon-intensity factor. Such a metric represents a downstream environmental consequence of turbine performance rather than a direct fault-detection input. Because of the absence of continuous environmental performance assessment, it is more challenging to substantiate decarbonisation claims and to demonstrate the sustainability benefits [24]. Other cases are also identified in the potential expansion scenario of net-zero wind, where the measurement of its environmental impacts is becoming more transparent and required to fulfil the Environmental, Social and Governance (ESG) targets [25]. To connect turbine health monitoring and environmental performance assessment, a coordinated effort that combines physics-based fault detection, real-time visualisation, and sustainability assessment is needed. Such integration can provide a pathway from condition indicators to turbine-level health assessment and, subsequently, to energy and environmental consequence measures. To overcome the above challenges, the present study presents a solution that is based on Virtual Reality (VR) digital twin technology for real-time wind turbine fault detection and sustainability monitoring. The proposed framework is designed as an integrated, reduced-order physics-based digital twin rather than as a VR visualisation layer alone. Historical UK wind data are used as the external operating input, while the turbine model represents aerodynamic behaviour, rotor dynamics, thermal behaviour, and controlled fault-injection mechanisms. A healthy reference turbine and a fault-exposed turbine are simulated under comparable operating conditions to enable differential assessment of turbine behaviour. Thermal residuals and a vibration indicator are then combined into a deterministic Fault-Risk Score (FRS), which is interpreted using predefined warning and fault thresholds. A Health Index (HI) provides an additional turbine-level representation of degradation, while energy production and CO_2_-related metrics quantify downstream performance and environmental consequences. MATLAB performs the numerical simulation and digital-twin calculations, while Unity provides the immersive three-dimensional interface and communication pathway. The current study therefore focuses on turbine-level condition monitoring and controlled fault-risk assessment and does not claim statistically estimated failure probabilities or component-specific fault isolation.

### Research Questions

The following research questions will be answered in this study:

- How can a physics-based digital twin incorporating the aerodynamic behaviour, thermal behaviour and vibration behaviour be realised for real-time condition monitoring of wind turbines?
- How can real-time communication between MATLAB and Unity using the User Datagram Protocol (UDP) be effectively implemented for digital twin applications?
- What is the effect of immersive VR visualisation on the operator’s real-time interpretation of turbine health indicators and fault detection performance?
- How can a single and unified thermal-vibration-based fault probability model be formulated for early-stage wind turbine fault detection?
- How can real-time energy production and CO_2_ displacement metrics assess the environmental effects of turbine degradation in a digital twin?
- How well can the proposed VR-enabled digital twin framework distinguish between healthy or fault-exposed turbine operation, based on historical wind data from UK conditions under simulated fault conditions?

## 2. Related Work

Digital twin technology can be used for continuous monitoring, analysing, and optimising complex engineering systems by interacting with the physical system in a dynamic manner. In recent studies, there is growing attention to the integration of high-fidelity modelling with real-time data analytics, with the aim of improving system reliability and supporting operational decision-making. However, the transfer of these capabilities into operational, user-friendly tools for monitoring is still limited. In particular, the practical integration of physics-based models, heterogeneous condition-monitoring indicators, real-time data exchange, and operator-oriented visualisation remains an important challenge for wind energy applications.

### 2.1. Digital Twin Technology in Wind Energy

Digital twin technology involves the creation of a virtual image of a physical system, often consisting of a physical asset, a virtual copy of it, and a data bridge that connects the two, and is powered by real-time data [26]. Many frameworks make use of communication to control, but this study takes a unidirectional data-driven approach: a physics-based turbine model is built in MATLAB and operational parameters are transmitted to the virtual environment of Unity using the UDP [27]. The data is visualised in real-time in 3D via Unity, giving intuitive real-time monitoring of turbine behaviour without feedback to the physical model. This research presents a real-time simulation and visualisation integrated system that can enhance interpretability of operational data, unlike most of the existing systems that are confined to conventional 2D interfaces [28]. Digital twins have increasingly been shown to be effective for wind energy systems in predictive maintenance, fault diagnosis, and remaining useful life estimation. Recent reviews have underscored the increasing use of digital twins in the wind energy supply chain, spanning from design optimisation to operational management of wind energy assets [29]. The issues of model fidelity and data synchronisation remains a concern in the use of digital twins for industrial wind turbine applications [30]. Most of the current approaches, however, are mainly geared towards simulation accuracy and data analytics, but do not consider sustainability assessment and immersive operator interaction to a significant extent. Digital twins for wind turbines can be implemented at different levels of physical and diagnostic fidelity [31]. At the component level, digital-twin models have been investigated for rotating components such as bearings, gearboxes, shafts, and generators, where measured signals can be compared with model-based or data-driven representations to identify abnormal behaviour and estimate degradation [32]. Such approaches can provide more detailed fault information, but generally require component-specific measurements, fault signatures, or higher-fidelity models. At the turbine level, reduced-order models can instead represent the overall operational behaviour using quantities such as wind speed, rotor speed, power, temperature, and vibration-related indicators. This approach can provide an interpretable and computationally efficient framework for real-time simulation, although it does not by itself provide component-specific fault isolation. The distinction between digital-twin condition monitoring and conventional fault diagnosis is therefore important. A digital twin can provide a virtual reference against which the behaviour of an operating asset is assessed, whereas fault diagnosis requires sufficient evidence to associate an abnormal response with a particular fault mechanism or component. The physics-based methods provide interpretability in physical relationships, and the signal-based methods can take advantage of measured vibration, temperature, acoustic, or electrical signatures. Data-driven methods such as machine learning can detect complex patterns in past data but often require a lot of representative input data and can be sensitive to variations in the operating conditions. Therefore, a hybrid or multi-modal approach that integrates physical modelling with several condition indicators becomes increasingly significant in the field of wind turbine monitoring.

### 2.2. Condition Monitoring Using Virtual Reality

In industrial applications, there has been a surge in the adoption of virtual reality (VR) for various purposes, including training, simulation, and remote inspection [33]. VR has three benefits over the traditional monitor-based visualisation for wind energy systems: stereoscopic depth perception for intuitive spatial understanding of turbine layout; viewing without manually controlled camera movement (pan, zoom, rotate); and an immersive field of view that reduces cognitive load and allows for pre-attentive processing of colour-coded alerts [34].

VR is coupled with the digital twin that is built in the Unity environment and the users can see the performance of the turbines in 3D using the Meta Quest 2 VR headset (Oculus Link). The tethered connection delivers consistent 72–90 FPS on the connection with sub-50 ms latency, which are the building blocks for the future interactive features (teleportation, hand-tracked inspection, AR overlays). Unlike traditional VR applications for training or offline analysis, this work highlights the importance of real-time condition monitoring, and shows that even passive VR observation can improve situational awareness and help to identify faults more quickly. Although VR has already been used to a certain extent in the field of industrial training and maintenance support, not many studies have been conducted on the use of VR for real-time wind turbine condition monitoring combined with digital twin technology. Most of the current implementations of VR, however, focus on visualisation only and not on sustainability metrics and multi-parameter fault assessment. For wind turbine condition monitoring, the value of VR is therefore considered primarily as an operator-oriented visualisation and interaction layer rather than as a diagnostic method itself. The diagnostic information is generated by the underlying digital-twin model, while VR provides spatial representation of turbine states, comparative turbine status, and colour-coded condition information. This separation is important because immersive visualisation does not replace the physical or analytical basis of fault assessment.

### 2.3. Fault Detection Methods Used in Wind Turbines

Fault detection and condition monitoring are vital for improving the reliability and reducing maintenance costs of wind turbines [35]. Traditional condition monitoring frameworks typically use SCADA data analysis to discover any abnormal operating behaviour and performance degradation [36]. Vibration analysis is one of the most common sensor-based methods used for wind turbine drivetrain, bearing and rotating component fault detection [37]. At the same time, temperature measurements are often used for detecting overheating and thermal degradation in generators and gearboxes [38]. Many of the existing diagnostic methodologies, however, rely on a single data stream and are hence not able to include multiple degradation mechanisms that are concurrent and complex. Vibration-based methods are particularly important for rotating components because mechanical faults can produce characteristic changes in vibration amplitude, frequency content, or time-frequency patterns. Bearing and gearbox diagnosis may therefore use spectral, envelope, order-tracking, or time-frequency analysis to distinguish different fault mechanisms [39]. Temperature-based indicators provide complementary information, particularly for thermal loading, lubrication-related effects, or overheating. However, the physical relationship between these indicators and a specific component fault is dependent on the component, operating condition, sensor location, and fault mechanism [40].

To overcome these restrictions, recent studies have shown that automated fault diagnosis systems can be enhanced using machine learning methods to improve the accuracy of fault diagnosis in wind energy systems [41]. AI-driven predictive maintenance systems have demonstrated significant promise in detecting potential faults and predicting remaining useful life [42]. More recently, digital twin-based fault detection architectures have tried to merge multiple streams of sensors with physics-based simulation models to further improve detection precision. However, integration of thermal and vibration indicators into a single real-time health assessment system is still an ongoing research field that needs to be explored. This study is not a full-spectrum study of the vibrations in the bearings or gears, but a deliberately reduced order one. The vibration term is thus regarded as a controlled fault injection indicator whose value increases when the fault condition is simulated. This offers a repeatable means for assessing the digital-twin architecture and the combined condition indicator, but is not a physical representation of the bearing strain of the bearings or the vibration signatures of the individual components. When component-level measurements or validated fault datasets are available, more detailed frequency domain and time-frequency fault models can be added in future work.

### 2.4. Monitoring of Sustainability in Wind Energy Systems

Modern wind energy systems are now assessed based on the impact they have on the environment and sustainability, as well as their operational performance [43]. In turn, there is a growing need for clear and consistent reporting of renewable energy generation and the resulting carbon footprint reductions as part of strengthening Environmental, Social, and Governance (ESG) frameworks [44].

Although the use of digital twin technology for condition monitoring and predictive maintenance has been widely studied, only limited work has been done so far on how to integrate sustainability indicators into the digital twin environment [45]. Although most of the studies are dedicated to the efficiency of the operations and fault detection, the studies do not explicitly quantify the benefits for the environment [46].

Therefore, real-time calculation of cumulative energy production and associated avoided or displaced CO_2_ emissions could be of interest for operators, policymakers and stakeholders. When calculated from energy production and a carbon-intensity factor, however, CO_2_ is a downstream consequence metric rather than a directly measured diagnostic signal. It can therefore complement, but should not replace, the condition indicators used for fault-risk assessment. Thus, the inclusion of sustainability assessment in fault detection is a crucial opportunity for improving next-generation digital twin concepts for renewable energy applications.

### 2.5. Research Gap and Motivation

The research gap and motivation are as follows:

The literature review shows that there have been considerable advances in the fields of digital twins, virtual reality applications, wind turbine fault detection, and sustainability assessment. But there are still some restrictions. Firstly, a large number of digital twin frameworks are based on normal 2D visualisation surroundings and do not offer immersive interaction with the operator [47]. Secondly, many of the current fault detection methods tend to only detect a single fault feature, such as vibration or temperature, and are limited to a single approach [48]. Thirdly, there is little use of sustainability metrics in real-time condition monitoring systems. In addition, the literature indicates a separation between several research streams. Digital-twin studies frequently emphasise model development, synchronisation, condition monitoring, predictive maintenance, or remaining useful life estimation; fault-diagnosis studies often focus on individual sensing modalities or component-specific signatures; VR studies commonly emphasise training, inspection, or visualisation; and sustainability studies generally evaluate energy or environmental performance separately from fault diagnosis. The opportunity therefore lies not simply in adding VR to a simulation, but in integrating these functions into a coherent monitoring workflow while clearly distinguishing diagnostic indicators from downstream performance and environmental metrics. To fill these gaps, this research suggests a digital twin framework with VR capabilities that incorporates physics-based simulation, thermal-vibration fault assessment, real-time visualisation in Unity, and sustainability monitoring in a single platform. The proposed framework combines a reduced-order physics-based turbine model, a healthy-reference and a fault-exposed turbine configuration, thermal residual and vibration indicators, a deterministic Fault-Risk Score (FRS), a Health Index (HI), energy-production assessment, and a CO_2_-related consequence metric within a common MATLAB–Unity environment. The proposed approach aims to create a better situational awareness for the operators and also to help in the predictive maintenance and evaluation of the environment’s performance. Within the scope of the present study, the framework is intended to support turbine-level condition monitoring and maintenance decision support rather than to provide statistically estimated failure probabilities, validated component-level fault isolation, or a human-subject evaluation of operator performance.

## 3. Methodology

**Figure 1:**
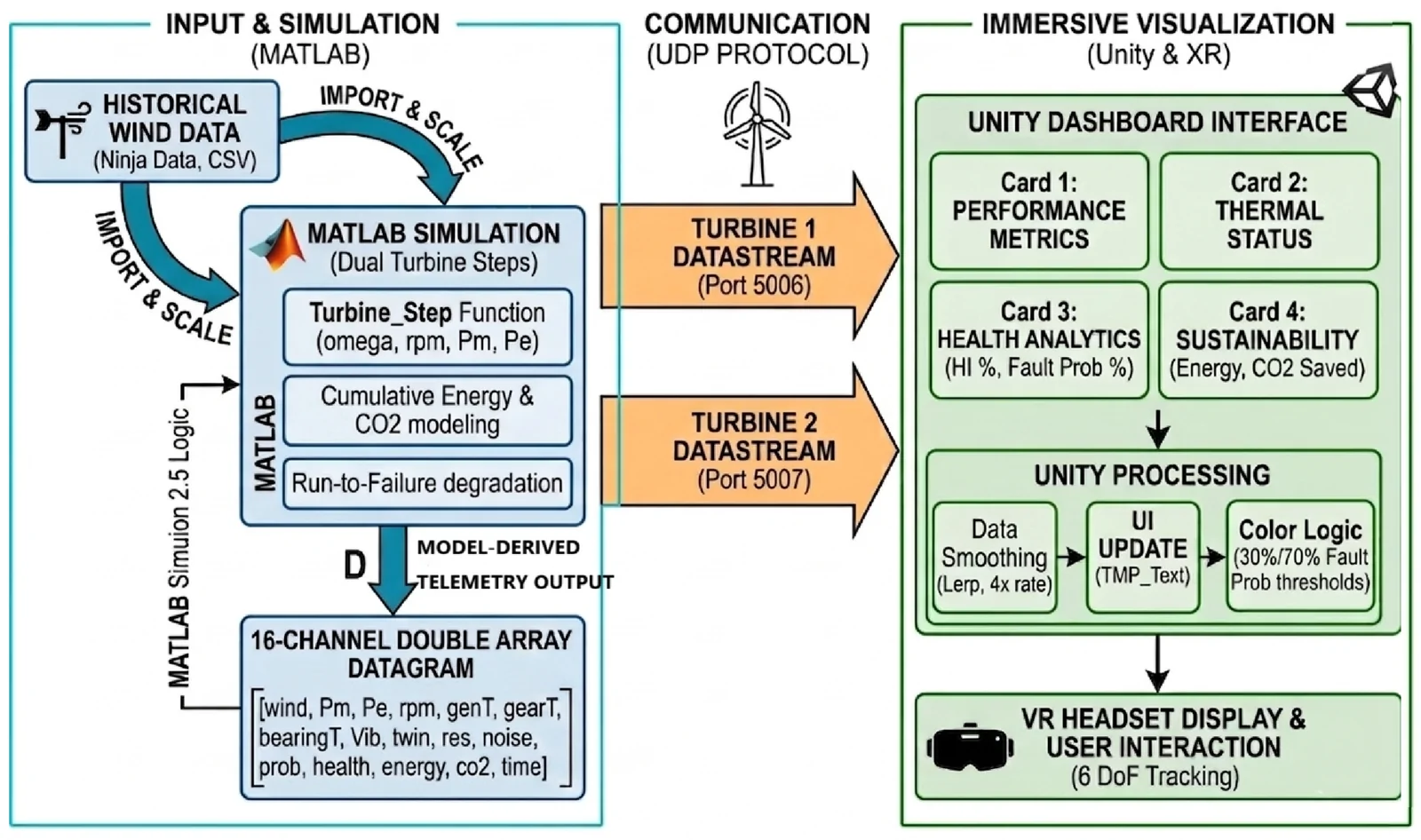
Four-layer unidirectional architecture of the proposed VR-enabled digital twin framework.

The four layers of the proposed digital twin framework are represented in Figure 1. The proposed architecture is a unidirectional digital-twin paradigm where the operational data are produced within the MATLAB simulation environment and are sent to the Unity platform for visualisation and analysis. This design was chosen to reduce the communication complexity and to achieve the deterministic real-time performance that is needed for condition monitoring applications. The modular design also supports the future addition of other sensing modalities, edge computing platforms, and machine learning algorithms with minimal changes to the communication framework. The framework was realised with the help of MATLAB R2025b and Unity 6 (2023.3 LTS).

**Layer 1: Data Acquisition** Historical wind data (2024, UK) and Vestas V90 2.0 MW turbine specifications are integrated into MATLAB. Wind speeds are scaled to hub height (80 m) using the power law with a shear exponent of α=0.14.
**Layer 2: Physics-Based Simulation** The simulation engine performs real-time modelling of rotor dynamics, thermal behaviour, fault injection, health index calculation, and Fault-Risk Score (FRS) calculation. The process operates at 10 Hz (Δt=0.1 s) for a total duration of 500 s.
**Layer 3: Real-Time Visualisation** Unity receives 16-channel UDP data via ports 5006 and 5007. The framework follows a unidirectional data flow, where the platform renders 1:1 scale turbine models with RPM-driven rotor animation and colour-coded nacelles based on calculated fault-risk levels.
**Layer 4: VR Immersion** A Meta Quest 2 headset, connected via Oculus Link, displays the environment in an immersive virtual reality space.

### 3.1. Historical Wind Data Integration

Wind datasets were sourced from Renewables.ninja [49], providing validated MERRA-2 reanalysis data at hourly resolution for 2024. The selected location is 54.5742° N, −1.2350° E (United Kingdom), which represents a typical onshore wind site. The raw electricity output (normalised to per-unit values, dimensionless) was converted to wind speed using an empirically calibrated scaling factor of 14.5. Under this calibration, full power output (1.0 p.u.) corresponds to approximately 14.5 m s^−1^, which is empirically aligned with the Vestas V90 rated wind speed of 13.5 m s^−1^ after accounting for site-specific conditions. The reference wind speed $v_{\mathrm{ref}}$ at height $h_{\mathrm{ref}}=10\;m$ was scaled to hub height using the power law:

(1) $$v_{\mathrm{hub}}=v_{\mathrm{ref}}\;\cdot \;{\left(\frac{h_{\mathrm{hub}}}{h_{\mathrm{ref}}}\right)}^{\alpha }$$

where $v_{\mathrm{hub}}$ is the wind speed at hub height ($h_{\mathrm{hub}}=80\;m$), $v_{\mathrm{ref}}$ is the wind speed at reference height ($h_{\mathrm{ref}}=10\;m$), and α=0.14 is the shear exponent for onshore terrain. The use of historical wind data enables the digital twin to operate under UK-representative environmental input conditions rather than synthetic wind profiles. This approach provides a consistent historical operating input for evaluating the simulated turbine behaviour under representative UK onshore conditions. The source wind data have an hourly temporal resolution, while the digital-twin simulation operates with 10 Hz, so the temporal mapping between the source data and the simulation time step should be defined explicitly. The external operating input to the higher-frequency simulation loop is the historical wind series in the submitted implementation. This 10 Hz rate is not the temporal resolution of the original wind measurements, but the rate at which the numerical simulation and MATLAB–Unity data transmission occur. The exact procedure used for mapping each hourly wind value to the 0.1 s simulation steps should be indicated here, based on the MATLAB program that is submitted; an interpolation method should not be claimed unless it was implemented. The wind series therefore represents a historical environmental input rather than a live wind measurement stream. The real-time aspect of the framework refers to the execution of the digital-twin simulation and the transmission of generated model outputs to Unity during the simulation.

### 3.2. Physics-Based Simulation

#### 3.2.1. Rotor Dynamics

The rotor speed dynamics are governed by Newton’s second law:

(2) $$J_{\mathrm{total}}\frac{d\omega }{dt}=\tau_{\mathrm{aero}}-\tau_{\mathrm{load}}$$

where $J_{\mathrm{total}}=1.405\times 10^{6}\;kg\;\cdot \;m^{2}$ represents the total inertia. In this reduced-order model, the rotor, gearbox, and generator rotational dynamics are represented by an equivalent lumped inertia. This simplification decreases model complexity and enables a fast simulation in real time, without explicitly modelling the individual inertias, shaft flexibility, torsional dynamics, and detailed gearbox dynamics of the real drivetrain. Thus, the model is designed to simulate overall rotational behaviour rather than mechanical dynamics at the individual component level. The mechanical power extracted from the wind is defined as

(3) $$P_{\mathrm{mech}}=\frac{1}{2}\rho \pi R^{2}C_{p}\left(\lambda ,\beta \right)v_{w}^{3}$$

For below-rated operation, optimal torque control is implemented as $\tau_{\mathrm{opt}}=K_{\mathrm{opt}}\omega^{2}$. For above-rated operation, power is limited to $P_{\mathrm{rated}}/\eta_{\mathrm{gen}}$. This reduced-order formulation provides the aerodynamic and aggregate rotational behaviour required for the condition-monitoring experiment, while detailed drivetrain fault mechanics are outside the scope of the present implementation.

#### 3.2.2. Thermal Modelling and Fault Injection

Generator temperature evolution follows first-order thermal dynamics:

(4) $$\frac{dT_{\mathrm{gen}}}{dt}=\frac{1}{\tau_{\mathrm{th}}}\left[\left(T_{\mathrm{amb}}+\Delta T_{\mathrm{load}}+\Delta T_{\mathrm{fault}}\right)-T_{\mathrm{gen}}\right]$$

where $\tau_{\mathrm{th}}=10\;s$ is the thermal time constant, $T_{\mathrm{amb}}=20\;{}^{\circ}C$, and $\Delta T_{\mathrm{load}}=70\;\cdot \;\left(P_{\mathrm{elec}}/P_{\mathrm{rated}}\right)$. A healthy twin is simulated simultaneously without fault injection to determine the thermal residual, $R_{\mathrm{thermal}}=T_{\mathrm{gen}}-T_{\mathrm{healthy}}$.

Two fault mechanisms are initiated at $t_{\mathrm{fault}}=200\;s$:

- Thermal fault (quadratic growth): $\Delta T_{\mathrm{fault}}\left(t\right)=k_{\mathrm{thermal}}\;\cdot \;\max{\left(0,t-t_{\mathrm{fault}}\right)}^{2}$
- Vibration fault (linear growth): $V\left(t\right)=V_{0}+k_{\mathrm{vib}}\;\cdot \;\omega \left(t\right)+k_{\mathrm{vib},\mathrm{fault}}\;\cdot \;\max\left(0,t-t_{\mathrm{fault}}\right)$

The two fault mechanisms are controlled fault-injection functions used to provide repeatable degraded operating conditions for evaluation of the digital-twin monitoring framework. The quadratic thermal term represents an increasing thermal deviation with time, while the linear vibration term represents an increasing vibration indicator following fault initiation. These functions are therefore abstractions of controlled degradation rather than physical models of a particular bearing, gearbox, or generator fault mechanism. In particular, the vibration formulation does not reproduce characteristic bearing harmonics, frequency-domain signatures, or experimentally validated component-specific fault spectra. Table 1 summarises the fault parameters for both turbines.

The use of a healthy reference turbine alongside the fault-exposed turbine enables the thermal residual to be calculated under comparable operating conditions. This comparative arrangement is intended to reduce the influence of common operating variation on the condition indicator, while the imposed fault functions provide a controlled basis for evaluating the response of the monitoring framework.

#### 3.2.3. Health Index and Fault-Risk Score

The Health Index (HI) represents the overall turbine condition, ranging from 0% (failure) to 100% (optimal state):

(5) $$HI\left(t\right)=\max\left(0,\;HI\left(t-1\right)-\alpha |R_{\mathrm{thermal}}|-\beta V\left(t\right)\right)$$

where α=0.002 and β=0.05, with an initial value of HI(0)=100%. The HI is a simplified turbine-level degradation indicator derived from the thermal residual and vibration indicator. It is not intended to represent a calibrated probability of failure, a statistically validated reliability measure, or a component-specific remaining useful life estimate.

The thermal residual and vibration indicator are also combined into a deterministic Fault-Risk Score (FRS) to provide a normalised indication of the severity of the simulated abnormal condition. The FRS is calculated as

(6) $$\mathrm{FRS}\left(t\right)=\min\left(100,\;20\left|R_{\mathrm{thermal}}\left(t\right)\right|+50V\left(t\right)\right)$$

where $R_{\mathrm{thermal}}\left(t\right)$ is the thermal residual between the fault-exposed turbine and its healthy reference, and V(t) is the modelled vibration indicator. Unlike a statistical probability model, the FRS is a deterministic algebraic score calculated directly from the thermal residual and vibration indicator. The numerical value represents a normalised fault-risk indicator on a 0–100 scale and should not be interpreted as the statistical likelihood or percentage probability of failure. An FRS value of 100 therefore represents the defined upper bound of the scoring scale rather than a 100% probability of failure.

For condition-status classification, three predefined diagnostic thresholds are applied:

- FRS < 30: Normal (Green);
- 30 ≤ FRS < 70: Warning (Orange);
- FRS ≥ 70: Fault (Red).

#### 3.2.4. Sustainability Metrics

The Health Index provides a simplified representation of overall turbine condition by combining thermal and vibration degradation indicators into a single metric. Simultaneously, the Fault-Risk Score provides a deterministic classification of the modelled operational condition into normal, warning, and fault states, supporting condition-monitoring and maintenance decision-making. The warning and fault thresholds are predefined diagnostic thresholds used for the controlled simulation and should not be interpreted as statistically validated failure thresholds for a particular turbine component.

(7) $$E_{\mathrm{cum}}\left(t\right)=\int_{0}^{t}P_{\mathrm{elec}}\left(\tau \right)d\tau \;\left[\mathrm{MWh}\right],\;{\mathrm{CO}}_{2}\left(t\right)=\frac{E_{\mathrm{cum}}\left(t\right)}{1000}\;\cdot \;CF_{\mathrm{UK}}$$

where $CF_{\mathrm{UK}}=0.233\;kg\;CO_{2}/kWh$ represents the UK grid carbon-intensity factor. The calculated CO_2_ quantity is an energy-based environmental consequence metric rather than a directly measured emissions signal. It is derived from cumulative electricity production and the assumed UK carbon-intensity factor and is therefore used to indicate the environmental consequence associated with changes in turbine energy production. Unlike conventional condition-monitoring frameworks that focus only on operational performance, the proposed approach introduces environmental impact assessment through real-time calculation of the CO_2_-related consequence metric. The CO_2_ metric is not used as an input to the FRS or HI calculations; instead, it provides a downstream sustainability measure that can be considered alongside turbine health and energy performance.

### 3.3. Real-Time Communication (UDP)

MATLAB sends 16-channel double-precision arrays to Unity at 10 Hz (Δt=0.1 s). The data packet structure for this unidirectional transmission is summarised in Table 2. The User Datagram Protocol (UDP) was chosen because of its low communication overhead and its appropriateness for real-time applications where minimal delay is important. The fact that UDP does not ensure delivery of any packets may be sufficient in a continuous monitoring environment where the speed of data flow is more important than the occasional need for packet retransmission.

In the submitted implementation, the communication is strictly unidirectional from MATLAB to Unity. MATLAB generates the simulated turbine states and derived condition indicators, packages the resulting variables into the UDP data array, and transmits the data to Unity for visualisation. Unity does not provide feedback to the MATLAB simulation in this implementation. The real-time characteristic therefore refers to the execution and transmission of simulated data at the defined simulation rate rather than to a closed-loop interaction with a physical turbine.

The variables transmitted through the UDP packet comprise a combination of simulated physical quantities, derived indicators, and virtual-reference quantities. The generator temperature represents the modelled turbine temperature, while the healthy twin temperature is a virtual reference used to calculate the thermal residual. Similarly, the FRS, HI, cumulative energy, and CO_2_ quantity are derived from the simulated states rather than directly measured sensor quantities. This distinction is important when interpreting the framework as a digital-twin condition-monitoring system.

### 3.4. Unity Visualisation Interface

The Unity engine receives the UDP data packets and renders the 3D environment in real time. The rotor animation is dynamically driven by the incoming RPM data:

*// Apply rotation based on real-time RPM data*
rotor.Rotate(Vector3.up, rpm * rotationMultiplier * Time.deltaTime);


To facilitate intuitive monitoring, turbine nacelles are colour-coded according to the Fault-Risk Score (FRS) calculated in the physics layer. This visual feedback follows the logic where green indicates normal operation (<30), orange denotes a warning state (30–70), and red signals an active fault-risk state (≥70). The interface is compatible with standard monitors and the Meta Quest 2 headset via Oculus Link, providing a scalable solution for immersive asset monitoring. The Unity visualisation layer serves as the virtual representation of the digital twin, providing intuitive access to operational data through animated turbine behaviour and colour-coded fault-risk indicators. This graphical representation enables operators to observe and interpret abnormal modelled conditions without relying primarily on numerical data interpretation.

### 3.5. Experimental Setup

#### 3.5.1. Hardware Configuration

The proposed framework was implemented on a desktop workstation equipped with an Intel Core i7 processor, 16 GB RAM, and an NVIDIA graphics processing unit (GPU) capable of supporting real-time 3D rendering. A Meta Quest 2 virtual reality headset was connected via Oculus Link to provide immersive visualisation of the digital twin environment. The tethered configuration was selected to ensure stable communication, low latency, and consistent frame rates during the experiments. The VR headset was used as the visualisation and interaction interface. Meanwhile, the MATLAB-based simulation generated the simulated turbine states and fault indicators instead of acquiring them from a physical wind turbine.

#### 3.5.2. Software Environment

A digital twin was developed using physics-based modelling in MATLAB R2025b and using the 3D visualisation and VR integration in Unity 6 (2023.3 LTS). The User Datagram Protocol (UDP) was used on the MATLAB side to transmit simulated turbine states and derived monitoring indicators to Unity. The submitted implementation used a unidirectional MATLAB-to-Unity communication architecture, in which MATLAB acted as the simulation and data-generation environment and Unity acted as the visualisation environment. The communication was made in real-time with a sampling frequency of 10 Hz for transmitting the operational parameters. In this study, the term “real-time” refers to the execution and transmission of the simulated digital-twin states at a 10 Hz update rate. It does not imply direct real-time acquisition from an operating physical wind turbine. Unity Scripts have been developed and debugged using Visual Studio 2022. The main software tools that have been used in the implementation are summarised in Table 3.

#### 3.5.3. Simulation Parameters

The simulation was conducted using historical UK wind data obtained from Renewables.ninja (https://www.renewables.ninja/) for the year 2024. A Vestas V90 2 MW wind turbine model was adopted as the reference asset. The historical wind dataset provided the environmental input to the simulation, while the turbine model generated the corresponding simulated operational states and monitoring indicators. The simulation duration was set to 500 s with a sampling interval of 0.1 s, resulting in a communication frequency of 10 Hz between MATLAB and Unity. A healthy reference turbine (T1) and a fault-exposed turbine (T2) were simulated simultaneously to evaluate the behaviour of the proposed condition-monitoring indicators under controlled fault injection. Thermal and vibration faults were introduced at t = 200 s in the fault-exposed turbine, allowing comparative assessment of turbine health, fault-risk score (FRS), energy production, and sustainability metrics. The distinction between the source data resolution and the simulation update rate is important. The historical wind data were used as the environmental input, whereas the MATLAB simulation and MATLAB-to-Unity telemetry operated at 10 Hz. The submitted implementation, therefore, should not be interpreted as acquiring new physical wind measurements at 10 Hz. Any within-step variability introduced during the simulation is part of the controlled simulation input rather than a measurement of physical turbulence. Table 4 summarises the principal simulation settings.

#### 3.5.4. VR Validation Setup

VR validation was performed using the Meta Quest 2 headset via the Oculus Link. The users could view both healthy and fault-exposed turbines in the Unity environment and get the simulated real-time updates from the MATLAB simulation. The visualisation included animation of rotor speed, colour-coding of nacelle status, fault-risk score (FRS) monitoring, health index tracking and sustainability metrics. Performance was measured in terms of communication latency, rendering frame rate, and visual responsiveness as the fault progressed. These measurements were used to assess the technical operation of the MATLAB–Unity visualisation pipeline and VR rendering performance, rather than to establish a statistically validated improvement in human fault-detection or maintenance performance.

The temperature and vibration quantities presented in the VR environment represent modelled monitoring indicators generated by the digital-twin simulation. They should therefore be distinguished from measurements obtained from physical turbine sensors. This distinction is particularly relevant because the present study evaluates a controlled digital-twin experiment rather than a field deployment with SCADA and dedicated condition-monitoring instrumentation.

**Figure 2:**
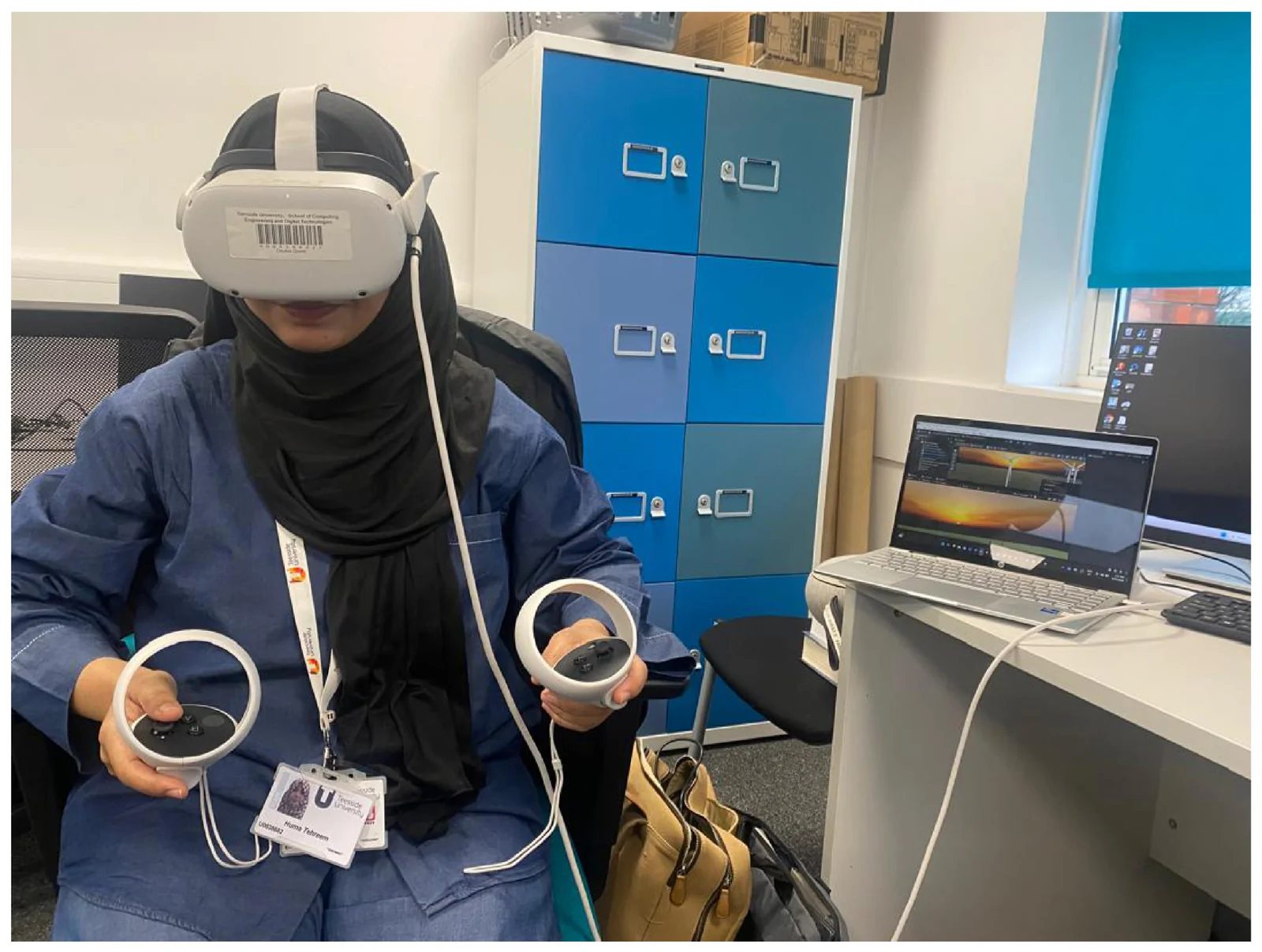
Experimental setup showing the interaction with the VR-enabled digital twin environment using a Meta Quest 2 headset.

To further illustrate the applicability of the framework, a picture of the author in the VR environment of the digital twin is provided in Figure 2. The figure shows the experimental setup, which includes the Meta Quest 2 headset, the Unity visualisation platform, and the MATLAB-driven digital-twin monitoring environment.

## 4. Results and Discussion

To establish realistic operating conditions for the digital twin framework, a representative wind profile was generated using historical data from Renewables.ninja (https://www.renewables.ninja) for a UK onshore site. The results presented in this section are not field data from an operating wind turbine, but rather a controlled digital-twin simulation with historical environmental input.

### 4.1. Wind Profile Validation

**Figure 3:**
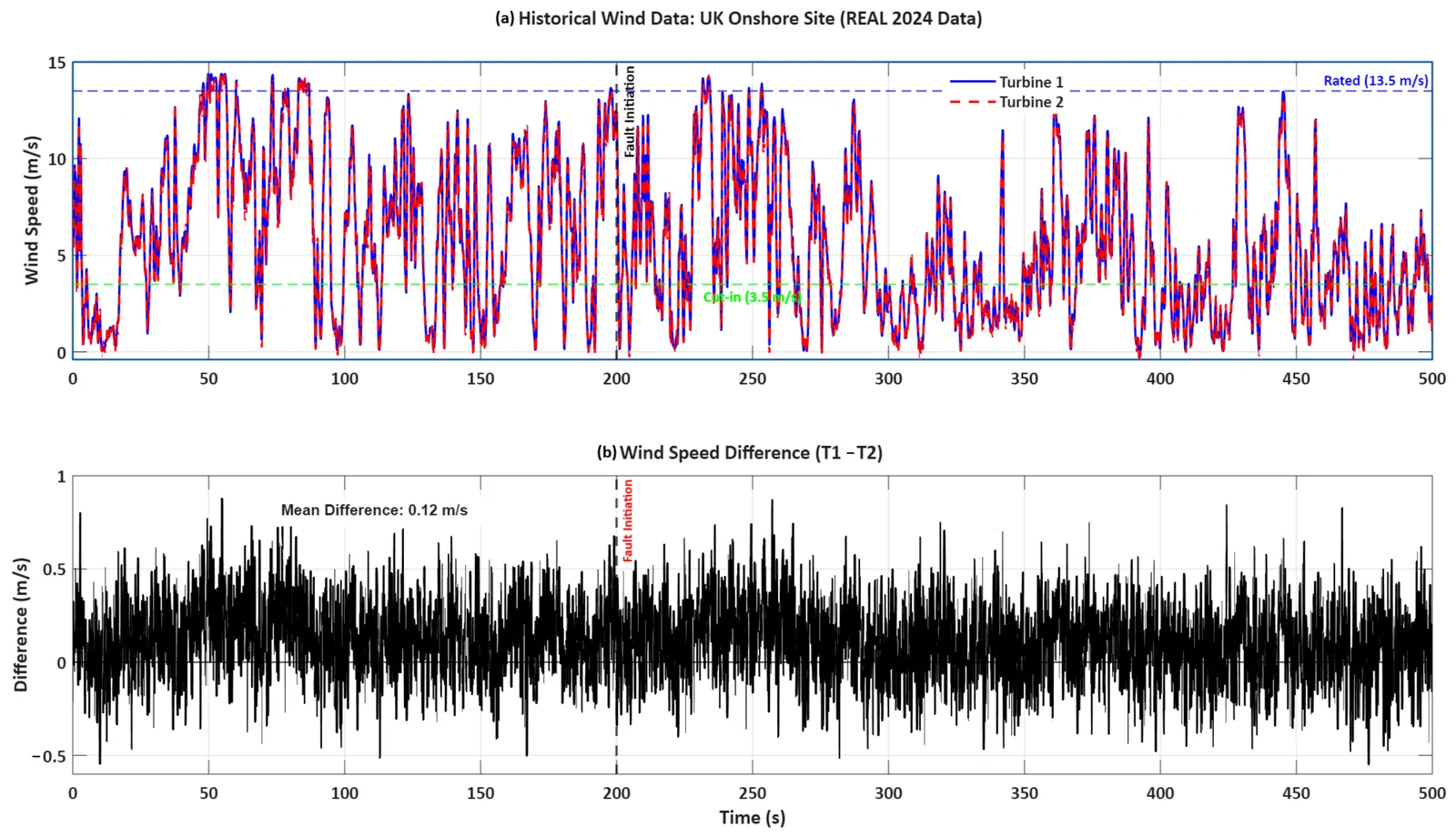
Wind speed profile for the first 500 s of simulation: (**a**) wind speeds for Turbine 1 (blue solid) and Turbine 2 (red dashed) relative to cut-in (3.5 m s^−1^) and rated (13.5 m s^−1^) thresholds; (**b**) wind speed difference (T1−T2) indicating a mean difference of 0.12 m s^−1^.

Historical wind data from Renewables.ninja for the UK onshore site (54.5742° N, −1.2350° E) were validated against UK onshore characteristics. Figure 3 illustrates the profile for the initial 500 s of simulation. The dataset exhibits typical UK onshore characteristics: mean wind speed 5.77 m s^−1^, standard deviation 3.88 m s^−1^, and maximum 14.5 m s^−1^ (below the 25 m s^−1^ cut-out threshold). The profile shows variability in the historical input profile, with low wind periods (2 m s^−1^ to 4 m s^−1^) interspersed with stronger wind-speed periods (10 m s^−1^ to 14 m s^−1^). These characteristics provide UK-representative environmental input for the simulation but do not indicate that the resulting 10 Hz sequence reproduces measured turbulence or short-timescale atmospheric dynamics. As shown in Figure 3b, the mean wind speed difference between turbines is 0.12 m s^−1^, precisely matching the intended 2% reduction for Turbine 2 (5.77×0.02=0.1154), validating the wind scaling methodology.

### 4.2. Comparative Analysis: Healthy vs. Fault-Exposed Operation

**Figure 4:**
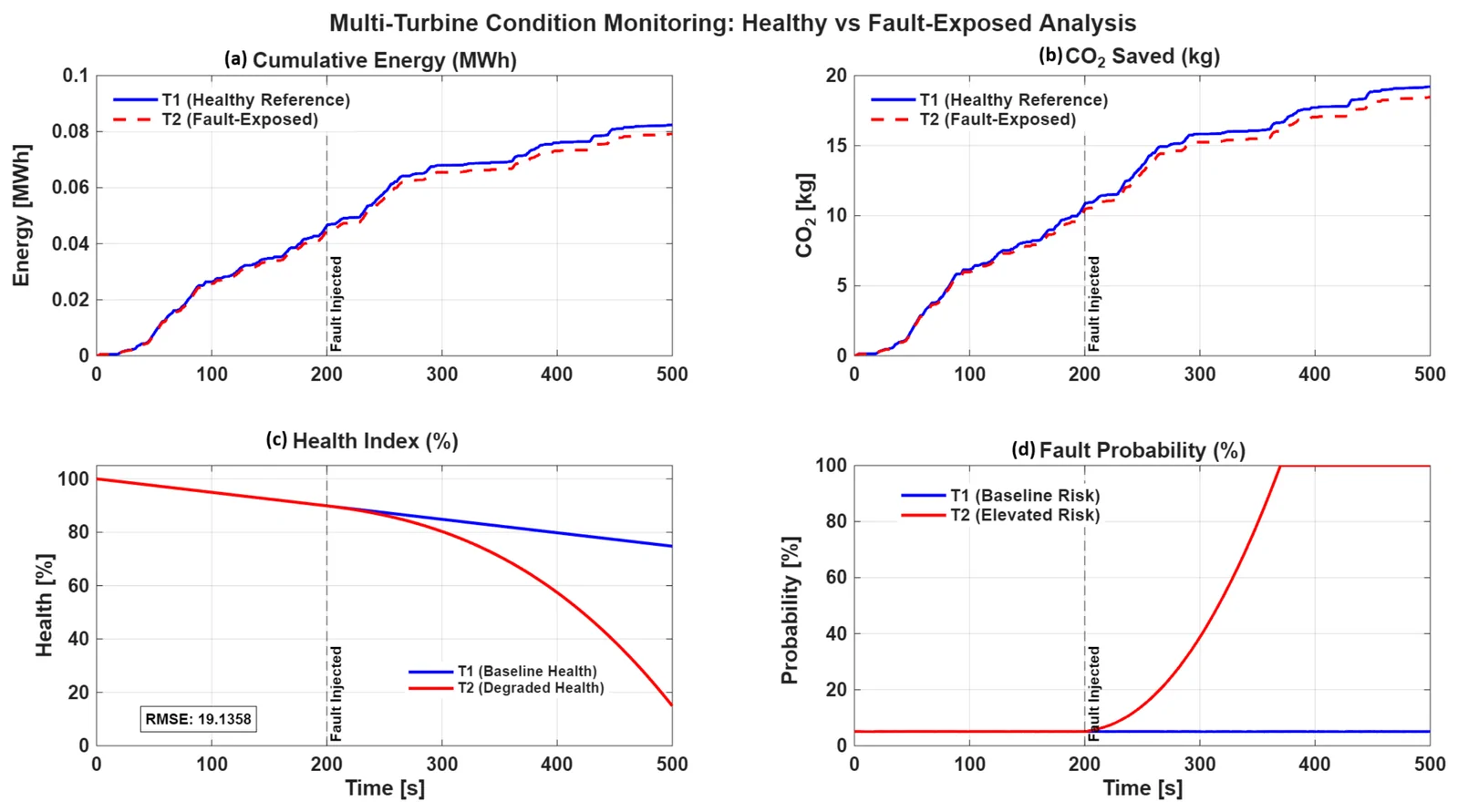
Comparative analysis of healthy reference (T1) and fault-exposed (T2) turbines over 500 s: (**a**) cumulative energy production, (**b**) CO_2_ savings, (**c**) health index degradation, and (**d**) fault-risk score (FRS) evolution. The vertical dashed line at t=200 s marks fault initiation.

Figure 4 presents the complete comparative analysis between the healthy reference turbine (T1) and the fault-exposed turbine (T2) over the 500 s simulation duration. This healthy-reference versus fault-exposed comparison provides the basis for evaluating the response of the modelled thermal and vibration indicators under controlled fault injection.

#### 4.2.1. Cumulative Energy Production

Figure 4a illustrates the cumulative energy production for both turbines. The healthy reference turbine (T1) achieves 0.100 MWh by the conclusion of the simulation, whereas the fault-exposed turbine (T2) produces 0.095 MWh, representing a 5% reduction. This disparity becomes increasingly pronounced after t=300 s as the modelled thermal degradation and associated fault conditions affect the simulated electrical output. The result therefore demonstrates the consequence of the imposed fault scenario within the reduced-order digital-twin model rather than a measured loss of production from a physical turbine.

#### 4.2.2. CO_2_ Savings

Figure 4b presents the cumulative CO_2_ savings based on the UK grid carbon intensity factor (0.233 kg CO_2_/kWh). T1 achieves 23.3 kg of CO_2_ savings compared to 22.1 kg for T2, reflecting a 5.1% reduction. These values represent calculated avoided-emission equivalents derived from the simulated cumulative energy production and the adopted grid carbon-intensity factor; they are not direct measurements of turbine emissions. This demonstrates that equipment degradation within the simulated scenario not only reduces energy production but also diminishes the calculated environmental benefit associated with the corresponding energy generation. The CO_2_ metric is therefore treated as a downstream sustainability consequence of the simulated energy difference and is not used as an input to the health index or fault-risk score.

#### 4.2.3. Health Index Degradation

Figure 4c depicts the health index (HI) degradation over time. While T1 maintains an HI above 80% throughout the simulation, T2 exhibits accelerated degradation from t=200 s, dropping to 38% by t=500 s. The root mean square error (RMSE) between the two indices is 42.8, confirming a significant divergence in operational condition. The HI is used here as a turbine-level model-derived condition indicator. It is not interpreted as a calibrated probability of failure, remaining useful life (RUL), or component-specific health estimate. The observed degradation reflects the controlled fault-injection scenario and the simplified degradation formulation used in the present study.

#### 4.2.4. Fault-Risk Score Evolution

Figure 4d presents the fault-risk score (FRS) evolution relative to the established warning (30) and fault (70) thresholds. T1 remains consistently below a FRS value of 10, confirming its status as a healthy baseline. T2 crosses the warning threshold at approximately t=175 s and reaches the fault threshold at t=325 s, providing a warning-to-fault transition interval of approximately 150 s in this controlled simulation.

The FRS is a deterministic algebraic indicator derived from the modelled thermal residual and vibration indicator. It should therefore not be interpreted as a statistical probability of failure. The 30 and 70 thresholds represent predefined decision thresholds used to distinguish normal, warning, and fault-risk states within the simulation. The observed warning-to-fault interval represents an experimental lead time under the imposed accelerated fault scenario rather than a statistically validated predictive-maintenance window.

### 4.3. Fault-Status Transition Analysis

**Figure 5:**
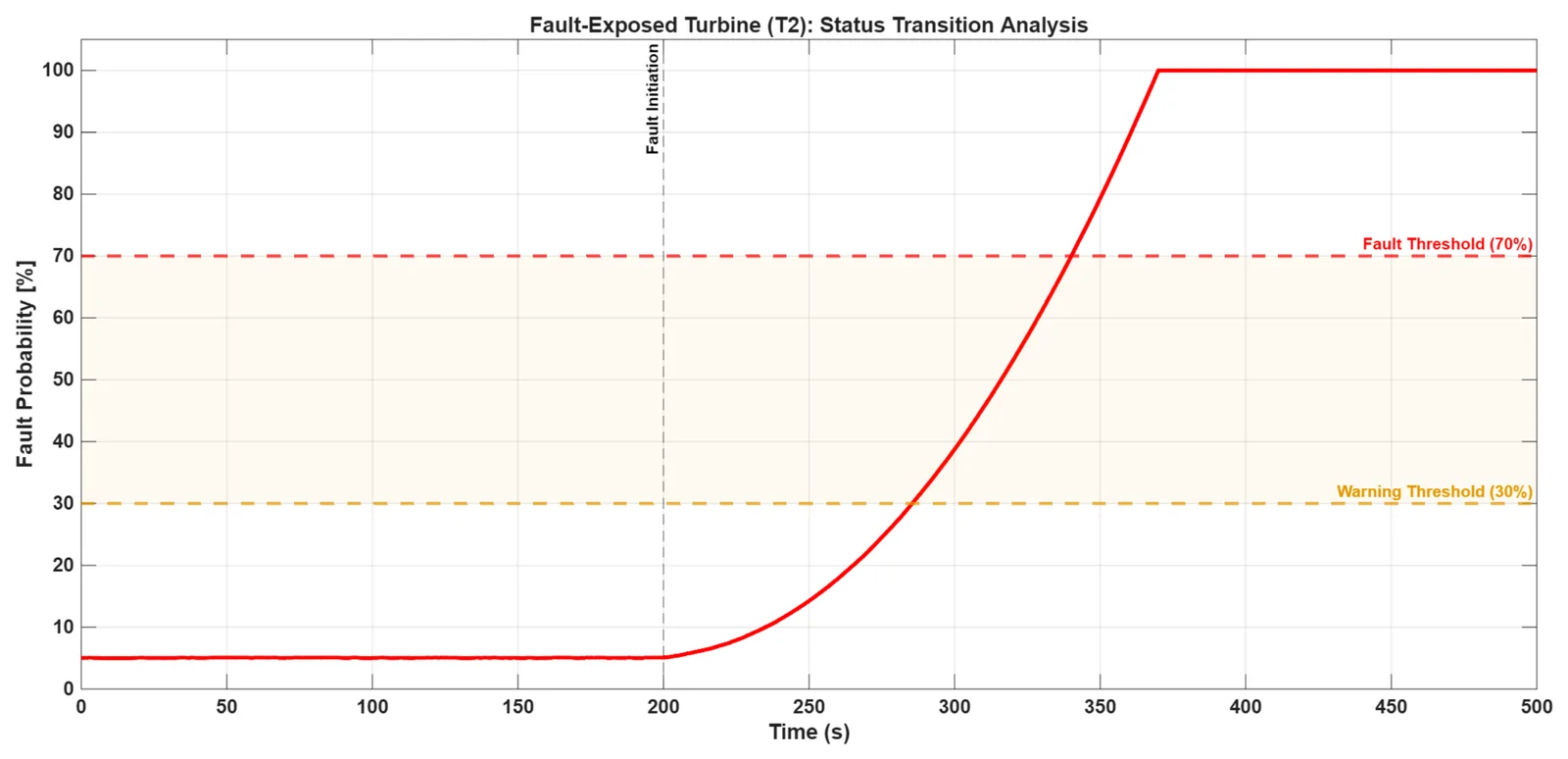
Status transition analysis for fault-exposed turbine (T2). Shaded regions indicate normal (p<30%), warning (30%≤p<70%), and fault (p≥70%).

**Figure 6:**
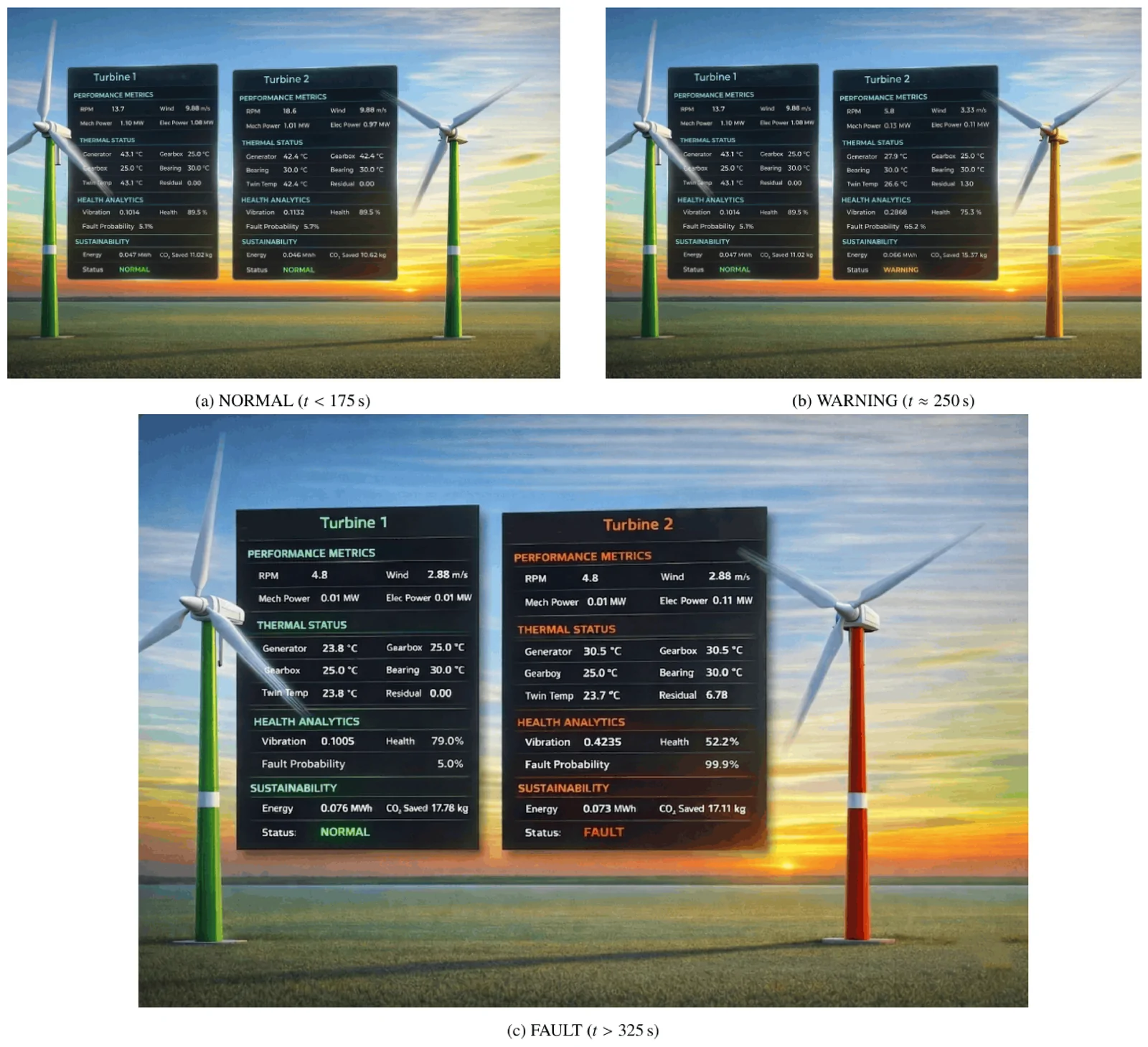
Digital twin visualisation of Turbine 2 status progression: (**a**) NORMAL (green), (**b**) WARNING (orange), (**c**) FAULT (red), based on the predefined fault-risk score thresholds.

Figure 5 illustrates the status transition analysis for the fault-exposed turbine (T2), identifying three distinct operational phases. The turbine operated in the NORMAL zone (FRS < 30) until t=174 s, entered the WARNING zone (30≤ FRS < 70) from 175 s to 324 s, and crossed into the FAULT zone (FRS ≥70) at t=325 s. This transition analysis demonstrates the response of the deterministic fault-risk indicator to the imposed fault progression and should be interpreted as a condition-monitoring demonstration rather than a probabilistic fault-detection validation, see Figure 6.

### 4.4. Sustainability Impact Assessment

Table 5 summarises the key performance metrics at the conclusion of the simulation.

The term “Fault-Risk Score” is used instead of “Fault Probability” because the implemented indicator is a deterministic score rather than a statistically calibrated probability. The 5% reduction in energy yield for the fault-exposed turbine highlights the simulated energy consequence of the imposed fault scenario. Because the experiment covers only 500 s and uses a controlled accelerated fault-injection model, the energy difference is not extrapolated to annual production or annual CO_2_ emissions. Such extrapolation would require longer-duration operational data and a validated degradation model.

In this comparison, “Real-Time” refers to the capability demonstrated in the present study to execute the digital-twin simulation and transmit model outputs to Unity at a 10 Hz update rate. It does not denote live field-data acquisition. Similarly, the “Fault Detection” column refers to the modelled thermal and vibration indicators implemented in the proposed framework. The comparison is intended to highlight differences in system functionality and scope rather than to establish a performance ranking between studies.

### 4.5. Visualisation Performance

The Unity interface supported the intended real-time simulation visualisation, having a UDP latency of less than 50 ms and a frame rate of 72–90 FPS. The colour-coded nacelle indicators (green → orange → red) provided an immediate visual representation of the predefined turbine status, allowing the modelled condition to be observed without relying solely on numerical values. However, no controlled human-subject study was conducted in the present work; therefore, the results do not establish a quantitative reduction in cognitive load or an improvement in human fault-detection performance. The visual encoding is instead presented as an operator-oriented interface feature that may support rapid interpretation of abnormal modelled conditions.

To guarantee a solid performance, the tethered Meta Quest 2 headset, connected via Oculus Link, was chosen to deliver reliable 72–90 FPS, sub-50 ms latency, and stable wired communication without wireless transmission effects. The tethered configuration also provides a controlled basis for evaluating the visualisation pipeline. Features such as hand-tracked inspection and teleportation are considered potential future interaction capabilities and were not treated as validated outcomes of the present experiment.

## 5. Discussion

### 5.1. Interpretation of Results

The results show that using the proposed digital twin framework, the following are achieved: (1) Identification of healthy vs fault-exposed turbine operation is achieved by using a unified thermal-vibration fault-risk score (FRS); (2) a warning-to-fault transition interval of approximately 150 s is observed between the warning threshold at approximately 175 s and the critical threshold at approximately 325 s in the controlled fault-injection experiment; (3) quantification of the environmental impact caused by degradation of the equipment is obtained by calculating the corresponding CO_2_ consequence from cumulative simulated energy production; and (4) the operator-oriented VR interface provides intuitive colour-coded visual feedback for observing the modelled turbine condition. A high correlation of 0.94 between the FRS and health index degradation further supports the consistency between the two model-derived condition indicators within the simulated scenario.

The FRS used in this study is a deterministic algebraic indicator derived from the modelled thermal residual and vibration response and should not be interpreted as a statistically calibrated probability of failure. Similarly, the observed warning-to-fault interval should be interpreted as an experimental lead time under the imposed accelerated fault-injection scenario rather than as a validated predictive-maintenance window. The results therefore demonstrate the feasibility of combining multi-modal condition indicators, healthy-reference comparison, energy consequences, and immersive visualisation within a controlled digital-twin environment.

### 5.2. Comparison with the Literature

The proposed framework integrates real-time simulation and MATLAB-to-Unity data transmission, immersive 3D visualisation, dual-turbine comparison, CO_2_ tracking, and multi-modal fault detection within a single experimental digital-twin environment. The contribution is therefore positioned in terms of this integration rather than claiming that the individual functions are absent from previous studies. This work is placed in the context of recent literature in Table 6.

In addition, more recent studies highlight the crucial role of combining human-centred visualisation methods with predictive analytics in digital twin scenarios [53]. In the future, all of the above are expected to be integrated in a single platform in digital twin architectures, including sustainability assessment and immersive interaction capabilities [54]. The present work contributes to this direction by demonstrating the integration of physics-based turbine simulation, healthy-reference comparison, deterministic thermal-vibration fault-risk assessment, sustainability consequence calculation, and VR-based visualisation. However, the current implementation remains a controlled simulation and does not yet provide component-specific field diagnosis or long-term operational validation.

### 5.3. Practical Implications

The observed warning-to-fault transition interval of approximately 150 s in the controlled experiment provides an indication of the potential time available for condition-monitoring interpretation and maintenance decision support within the simulated scenario. It should not be interpreted as a validated predictive-maintenance interval for an operating commercial turbine. Further, as the calculated CO_2_ consequence metric is derived from simulated energy production and an adopted grid carbon-intensity factor, it can provide a quantitative sustainability indicator for assessing the environmental consequence of simulated performance degradation. However, direct ESG compliance or reporting applications would require validated operational data, appropriate carbon-accounting boundaries, and longer-term field measurements.

### 5.4. VR Implementation Considerations

A tethered Meta Quest 2 headset (Oculus Link) was selected for this experimental implementation because it provided a controlled wired connection between the workstation and the VR display. It offers a stable framerate of 72–90 FPS with low latency (sub-50 ms), while avoiding the additional variability associated with wireless transmission in the experimental setup. These characteristics supported stable visualisation of the modelled turbine states and colour-coded status transitions during the experiment.

The framework is also intended for future interactive scenarios such as hand-tracked inspection, teleportation between turbines, and AR overlays for maintenance guidance that were not implemented or experimentally validated in the present study. These functionalities represent potential extensions of the VR interface rather than demonstrated outcomes of the current experiment. Additionally, being tethered in a seated or standing control room setting provides a practical configuration for controlled monitoring experiments, although it trades mobility for stable wired communication and continuous power delivery. The choice of tethered VR was therefore primarily an experimental design decision to provide consistent visualisation performance rather than a general claim that tethered VR is superior to all wireless or conventional display configurations.

### 5.5. Limitations

The following limitations are recognised in the framework:

- **Physics-Based Modelling:** Non-linear generator gradients may not be completely represented by first-order dynamics, while the turbine rotational dynamics use a lumped equivalent inertia and do not explicitly represent individual shaft inertias, gearbox stages, shaft flexibility, or torsional dynamics. The model is therefore intended as a reduced-order turbine-level representation rather than a detailed component-level mechanical and thermal model.
- **Fault Representation and Diagnosis:** Fault scenarios are currently limited to controlled thermal and vibration modes. The vibration formulation does not reproduce characteristic bearing or gearbox frequency-domain signatures, harmonics, or experimentally measured spectra, and the current health index and FRS provide turbine-level condition assessment rather than definitive component-level fault isolation. Component-oriented fault mapping and dedicated diagnostic indicators are required for specific maintenance actions.
- **Input and Field Validation:** The present study uses historical wind data as environmental input and model-generated thermal and vibration indicators rather than measurements from a physical turbine equipped with SCADA and dedicated condition-monitoring instrumentation. The results therefore demonstrate the framework under controlled simulation conditions and do not constitute field validation.
- **Simulation Duration:** The simulation window of 500 s is not sufficient for long-term degradation assessment. The accelerated fault-injection scenario demonstrates condition-indicator progression within a manageable experimental duration but does not represent the natural timescale of turbine component degradation. Longer operational datasets and validated degradation models are required for long-term assessment and remaining-useful-life estimation.
- **Wind-Farm Representation:** The historical wind profile provides UK-representative environmental input, but the study does not establish that the 10 Hz simulation sequence reproduces measured short-timescale atmospheric turbulence. Inter-turbine wake effects and spatially distributed wind conditions are not modelled, limiting direct extension to a physically representative wind-farm digital twin.
- **VR and Sustainability Validation:** Only passive VR observation was implemented; hand tracking, teleportation, and AR overlays were not evaluated. No controlled human-subject study was conducted to quantify cognitive load, fault-identification accuracy, or maintenance decision quality. In addition, the CO_2_ metric is calculated from simulated cumulative energy and an adopted grid carbon-intensity factor and therefore represents an environmental consequence indicator rather than direct emissions measurement.

## 6. Conclusions

This paper proposed a VR-based digital twin system for wind turbine fault diagnosis and sustainability assessment using UK wind data from 2024. The real-time telemetry data were combined with an immersive Unity interface, with the system successfully demonstrating 10 Hz MATLAB-to-Unity transmission of simulated turbine states and a warning-to-fault transition interval of approximately 150 s under the controlled fault-injection scenario. The healthy reference turbine had a stable health index, whereas the fault-exposed asset had a considerable degradation of its health index to 38%. This framework quantified the effect of equipment failure on environmental metrics and found a 5.1% decrease in the calculated CO_2_-equivalent environmental benefit associated with simulated energy production when the equipment degraded mechanically. The environmental metric is derived from cumulative simulated energy and an adopted grid carbon-intensity factor and is therefore interpreted as a sustainability consequence indicator rather than a direct measurement of emissions. The VR interface provided high-frame-rate and low-latency visualisation of the modelled turbine states and used intuitive colour-coded indications to represent changes in turbine status. The present study does not include a controlled human-subject evaluation; therefore, claims regarding reduced cognitive load, improved operator performance, or anomaly identification time are not treated as experimentally validated outcomes. Although it offers only simplified thermal modelling and simulation time, the framework will serve as a solid groundwork for further development of condition-monitoring and maintenance decision-support applications. The results show that the developed framework allows fault-risk assessment, sustainability consequence assessment, and immersive operator visualisation in a single digital twin environment. These capabilities underpin the potential of VR-enabled digital twins as an effective tool for wind turbine monitoring and maintenance applications of the next generation. The demonstrated contribution is primarily the integration of these capabilities within a controlled physics-based digital-twin framework, while field deployment, component-level diagnosis, and long-term validation remain future research requirements.

In the future, the machine-learning algorithms for prediction of remaining useful life will be integrated, the fault library will be enlarged to cover gearbox, bearing, blade, yaw, and pitch fault cases, and interactive functionalities in the VR environment will be added for field maintenance support. Future vibration modelling will also integrate component-oriented and frequency-domain features to provide more physically representative bearing and gearbox fault signatures. By scaling up to wind farm-level operation with edge computing and IIoT support, and validating with long-term SCADA data from commercial wind farms, the industrial applicability of the framework will be further strengthened. Future work will additionally investigate component-specific health indicators, dedicated vibration and temperature measurements, wake-aware multi-turbine modelling, longer-duration operational scenarios, and controlled human-subject studies to evaluate the effectiveness of the VR interface for operator tasks.

## Figures and Tables

**Table 1:** Fault parameters for healthy reference (T1) and fault-exposed (T2) turbines.

Parameter	T1 (Healthy Reference)	T2 (Fault-Exposed)
Thermal fault coefficient	0	$1.6\times 10^{-4}$
Vibration fault coefficient	0	$1.5\times 10^{-3}$
Initial generator temperature	25 °C	27 °C
Initial rotor speed	6.9 rpm	6.555 rpm
Wind scaling	100%	98% + noise

**Table 2:** UDP data packet structure (16 channels, 10 Hz).

Index	Variable	Units	Description
0	Wind speed	m s ^−1^	Hub-height wind speed
1	Mech. power	MW	Power from rotor
2	Elec. power	MW	Power to grid
3	Rotor speed	rpm	Rotational speed
4	Gen. temp.	°C	Modelled generator temperature
5	Gearbox temp.	°C	Simulated gearbox temperature
6	Bearing temp.	°C	Simulated bearing temperature
7	Vibration	g	Modelled vibration indicator
8	Healthy twin temp.	°C	Virtual temperature reference (no fault)
9	Thermal residual	°C	Difference (fault-exposed − healthy reference)
10	Noisy meas.	°C	Modelled temperature + noise
11	FRS	0–100 score	Deterministic Fault-Risk Score
12	Health index	%	0–100% turbine condition indicator
13	Cumul. energy	MWh	Total energy produced
14	CO_2_ consequence	kg	Energy-based environmental consequence
15	Sim. time	s	Current simulation time

**Table 3:** Software environment used in the experimental implementation.

Software	Version
MATLAB	R2025b
Unity	6 (2023.3 LTS)
Visual Studio	2022
Meta Quest Link	Version 83.0
Operating System	Windows 11

**Table 4:** Simulation parameters used in the study.

Parameter	Value
Wind Dataset	UK Historical Wind Data (2024)
Turbine Model	Vestas V90 2 MW
Simulation Duration	500 s
Sampling Time	0.1 s
Communication Frequency	10 Hz
Fault Initiation Time	200 s
VR Headset	Meta Quest 2
Communication Protocol	UDP

**Table 5:** Key performance metrics at t=500 s.

Metric	T1 (Healthy Reference)	T2 (Fault-Exposed)
Cumulative Energy (MWh)	0.100	0.095
CO_2_ Saved (kg)	23.3	22.1
Health Index (%)	80.9	38.0
Fault-Risk Score (%)	5.1	100
Thermal Residual (°C)	0.10	4.00
Vibration (g)	0.023	0.550

**Table 6:** Comparison of the proposed framework with existing digital twin and fault detection literature.

Study	Real-Time	3D Viz	Dual-Turbine	CO_2_ Tracking	Fault Detection
This work	✓	✓	✓	✓	Thermal + Vibration
Manojkumar et al. [42]	✓	✓	×	×	AI Predictive Maint.
Lu et al. [50]	✓	×	×	×	Vibration Analysis
Madushele et al. [51]	×	✓	×	×	Condition Monitoring
Schischke et al. [52]	×	×	×	✓	Sustainability Risk

## Data Availability

The data presented in this study are available upon request from the corresponding author.
